# A Novel EEG-Based Assessment of Distraction in Simulated Driving under Different Road and Traffic Conditions

**DOI:** 10.3390/brainsci14030193

**Published:** 2024-02-21

**Authors:** Vincenzo Ronca, Francois Brambati, Linda Napoletano, Cyril Marx, Sandra Trösterer, Alessia Vozzi, Pietro Aricò, Andrea Giorgi, Rossella Capotorto, Gianluca Borghini, Fabio Babiloni, Gianluca Di Flumeri

**Affiliations:** 1 Department of Computer, Control, and Management Engineering, Sapienza University of Rome, 00185 Rome, Italy; pietro.arico@uniroma1.it (P.A.); rossella.capotorto@uniroma1.it (R.C.); 2 BrainSigns SRL, 00198 Rome, Italy; alessia.vozzi@uniroma1.it (A.V.); andrea.giorgi@uniroma1.it (A.G.); gianluca.borghini@uniroma1.it (G.B.); fabio.babiloni@uniroma1.it (F.B.); gianluca.diflumeri@uniroma1.it (G.D.F.); 3 DeepBlue SRL, 00185 Rome, Italy; francois.brambati@dblue.it (F.B.); linda.napoletano@dblue.it (L.N.); 4 Virtual Vehicle Research GmbH, 8010 Graz, Austria; cyril.marx@v2c2.at (C.M.); sandra.troesterer@v2c2.at (S.T.); 5 Department of Anatomical, Histological, Forensic and Orthopaedic Sciences, Sapienza University of Rome, 00185 Rome, Italy; 6 Department of Molecular Medicine, Sapienza University of Rome, 00185 Rome, Italy; 7 College of Computer Science and Technology, Hangzhou Dianzi University, Hangzhou 310005, China

**Keywords:** EEG, attention, distraction, simulated driving

## Abstract

The drivers’ distraction plays a crucial role in road safety as it is one of the main impacting causes of road accidents. The phenomenon of distraction encompasses both psychological and environmental factors and, therefore, addressing the complex interplay contributing to human distraction in automotive is crucial for developing technologies and interventions for improving road safety. In scientific literature, different works were proposed for the distraction characterization in automotive, but there is still the lack of a univocal measure to assess the degree of distraction, nor a gold-standard tool that allows to “detect” eventual events, road traffic, and additional driving tasks that might contribute to the drivers’ distraction. Therefore, the present study aimed at developing an EEG-based “Distraction index” obtained by the combination of the driver’s mental workload and attention neurometrics and investigating and validating its reliability by analyzing together subjective and behavioral measures. A total of 25 licensed drivers were involved in this study, where they had to drive in two different scenarios, i.e., City and Highway, while different secondary tasks were alternatively proposed in addition to the main one to modulate the driver’s attentional demand. The statistical analysis demonstrated the reliability of the proposed EEG-based distraction index in identifying the drivers’ distraction when driving along different roads and traffic conditions (all *p* < 0.001). More importantly, the proposed index was demonstrated to be reliable in identifying which are the most impacting additional driving tasks on the drivers’ distraction (all *p* < 0.01).

## 1. Introduction

### 1.1. Human Distraction in Automotive

Driver distraction has emerged as a critical issue with profound implications for road safety. In this regard, it has to be noted that the most recent report provided by the World Health Organization [1] listed distracted driving among the most relevant causes of road accidents. Furthermore, even though traffic accidents have been acknowledged as a public health issue for many years, improvements and counteractions to this concern at a global level appear to be not enough, particularly concerning low and middle-income countries, as indicated by Tavakkoli et al. [2]. In this regard, scientific works such as the ones proposed by Oviedo-Trespalacios and Watson [3], Oviedo-Trespalacios et al. [4], and Oviedo-Trespalacios et al. [5] have provided empirical evidence highlighting the escalating threat to individuals’ health and road safety posed by distracted driving. This phenomenon encompasses both internal (psychological) and external (environmental) factors that can impact and impair the driver’s attention. Naumann and Dellinger [6] asserted that accidents resulting from distraction are more likely to lead to fatalities or severe injuries, a claim supported by statistics. In Europe, approximately 18% of all car accidents are attributed to distracted driving, with 9% of these incidents resulting in fatalities and an additional 18% causing serious injuries [1]. In this regard, various studies emphasize the importance of addressing road distractions in the formulation of policies aimed at enhancing road safety [7,8,9].

From a psychological point of view, contemporary perspectives underscore the increasing complexity of distracted driving. Gazder and Assi [10] categorize distraction into three types: manual, visual, and cognitive, with each type exacerbated by critical factors like the improper use of information and communication technologies, especially mobile phones, while driving [11,12]. Simultaneously, enduring sources of distraction, considered “traditional”, including billboards, road obstacles, challenging weather conditions, and the driver’s internal thoughts, continue to be prevalent [13,14,15]. This last aspect is extremely coherent with the recent technological development that characterized the automotive context. As vehicles become increasingly equipped with advanced infotainment systems, navigation tools, and communication devices, the potential for distraction intensifies. Beyond the immediate danger, the societal and economic impact of distracted driving encompasses medical costs, property damage, and loss of life.

Addressing the complex interplay of factors contributing to human distraction in automotive environments is crucial for developing effective interventions and technologies that promote safer driving habits and mitigate the toll of distracted driving on individuals and communities alike.

### 1.2. Psychological Definition of Distraction

Attention and distraction are intertwined, and understanding the latter requires first defining the former.

Attention is defined as the set of cognitive mechanisms that enable the selection and filtering of stimuli and the processing of information to provide an appropriate response output [16]. The object of attention can either be environmental stimuli actively processed by sensory systems or associative information and response alternatives generated by ongoing cognitive activity. Attention is a function highly associated with the level of psychophysiological activation.

Different types of attention can be observed: sustained attention, divided attention, attentional control, spatial attention, alertness, and vigilance [17,18]. This is a key point since it allows one to differentiate attentional functioning according to tasks that are performed.

Two relatively independent aspects relating to the attention span can be distinguished:

- Selectivity: attention may be *focused*, e.g., it may be centered on the color of a road sign, or *divided*, i.e., simultaneously directed at several eventsIntensity: attention may be considered *alertness*, e.g., put in operation when stopped at a red light; and *sustained* (*vigilance*), which allows one to continue to respond in a reasonable manner during the period in which a series of events may appear in an unforeseen manner.

However, these aspects are not working independently. The subject can flexibly adapt the use of these strategies to the situation by performing a conscious monitoring of the activity, enabling it to direct the attention to different aspects of the situation or to voluntarily organize the sequence of actions to be performed. This system is called Supervisory attentional control [17]. This type of control modulates all other attention processes.

On the other hand, distraction is examined across a variety of fields, and each one uses a slightly different definition. However, all the definitions share the presence of a main task where attention is focused, and where one or more distractions are present.

Distractions are usually differentiated between other secondary tasks (multitasking), external stimuli, and unrelated thought processes [19]. In the road transport mode, driver distraction is a well-studied instance [20]. In such a paradigm, a secondary task is often carried out concurrently with driving, while driving retains primacy. But, why is it difficult to perform two different tasks at the same time and divide attention?

- Structural interference: if two tasks share the use of the same processing mechanism or require the same processing stage, attention will scarcely be divided due to physical and cognitive structural constraints, and one of the tasks will be identified as a distraction.
- Resource interference: non-automatic mental operations require a certain number of attentional resources, and the task that receives the residual resources is identified as the secondary task. For instance, even if the secondary task does not require looking away from the road, it is possible that it will reduce driving performance given the modulation of attention and cognitive resources between two or more tasks.

A second type of distraction occurs when external stimuli unrelated to the task are perceived alongside the task. Despite their irrelevance, these stimuli have a negative impact on performance [21] as they require the same sensory systems needed to perform the task (i.e., structural interference) leading either to visual, manual, or auditory distractions or their possible combinations, and/or require dividing attentional resources to elaborate and plan how to behave based on the perceived stimuli (i.e., resource interference).

Lastly, the third type of distraction, mind-wandering, has no direct connection to a person’s current goals or environment [22] as was the case for multitasking or external stimuli. Mind-wandering is frequently defined as ‘‘task-unrelated thinking” [23]. Generally, these lapses of attention are observed during very monotonous tasks where there are low cognitive demands—such as driving in familiar situations [19].

One of the main challenges in investigating cognitive distraction and mind-wandering is lacking the capacity to directly measure the constructs. Conversely, the benefit of investigating, e.g., visual, manual, and auditory distractions is having the possibility to measure them by using quantitative and behavioral data gathering tools (e.g., eye-tracker, system performance data, etc.).

The present research aims at evaluating the neurophysiological correlates of cognitive distraction through the application of neurotechnologies. In this regard, the presented approach foreseen the selection of a wearable EEG system equipped with dry-based electrodes, in order to be compliant with the wearability and comfort requirements that characterize the automotive environment. The neurophysiological perspective on distraction will thus be discussed in the upcoming section.

### 1.3. Neurophysiological Characterization of Distraction: State of the Art

Once the psychological pillars of the distraction are defined, the state of the art of its neurophysiological characterization must be described. As clearly mentioned in the previous paragraph, the concept of distraction results in the combination of different factors under different dimensions. Therefore, the neurophysiological characterization of distraction needs to be compatible with such a definition. Over recent years, different scientific works have been focused on distraction detection through the processing of neurophysiological data. In this regard, it has to be noted that also subjective and behavioral measurements were largely explored in the scientific literature for distraction evaluation [24,25,26]. However, such approaches are susceptible to bias and lack objectivity. In fact, it is important to be aware of the pertinent limits of such evaluations due to their inherent subjectivity and inability to capture the “unconscious” process of underlying human behavior. Therefore, the neurophysiological-based approach results in a robust candidate for the objective evaluation of the distraction. In this regard, different research proposed the detection of cognitive distraction through the computation of Electroencephalography (EEG)-based features. For example, Perera and colleagues [27] proposed to employ specific brain-connectivity estimators through the EEG signal analysis for detecting the drivers’ distraction in a simulated environment. Similarly, Beltrán and colleagues [28] investigated the reliability of EEG-related features for evaluating human distraction in a realistic environment, as well as spectral EEG features were validated to be reliable indicators for the users’ distraction level among different operational environments [29]. Recently, machine learning-based approaches emerged to be reliable methodologies for distraction detection [30,31,32], especially in automotive contexts in which different kinds of data are usually available, such as behavioral, vehicle data, and physiological and neurophysiological data. The above-mentioned recent scientific works underlined how a distracted state while driving can be associated with specific spectral EEG feature variations, mainly focused on the theta and beta EEG frequency bands over the frontal and parietal EEG channels [28,29].

In any case, as briefly described, there is not a univocal measure to assess the degree of distraction, nor a gold-standard tool that allows to “detect” eventual events of distraction or the impact of specific road conditions and additional tasks over the driver’s attention. This represents the relevant research gap to which the present research aimed to address.

### 1.4. Objective of the Presented Study

Given the above-mentioned aspects related to the wide impact of human distraction in automotives, and the lack of a specific assessment of human distraction in such a context paired with an objective methodology for its evaluation in terms of the different declinations of driving distractions in realistic environments through a unique indicator, the present study aimed at

(i) developing an EEG-based “Distraction index” obtained by the combination of the driver’s mental workload and attention neurometrics on the basis of EEG data coming from an experimental study in simulated driving settings under different road and traffic conditions;
(ii) investigating and validating its reliability by analyzing together subjective (i.e., self-assessment) and behavioral (driving parameters and ocular movements) measures coming from the participants themselves.

## 2. Materials and Methods

### 2.1. Participants

Twenty-five (25) participants in possession of their driving license, with normal or corrected-to-normal vision, were recruited on a voluntary basis. The participants were selected in order to have a homogeneous sample in terms of age (age ranged from 18 to 69 years old, corresponding to an average of 26 years old), driving experiences, and cars that they normally used to drive. With a distribution of 14 male and 11 female participants, gender distribution was largely equal. All the involved participants were in healthy conditions and did not present any histories of psychiatric neurological diseases, medication, drug abuse, or visual and auditory acuity. A written informed consent was obtained from each participant after an explanation of the study. The experiment was conducted following the principles outlined in the Declaration of Helsinki of 1975, as revised in 2000. To respect the privacy of participants, only aggregate results will be reported, and any results based on a single identity analysis will be presented.

The experimental procedure was designed in order to require the participants to perform simulated driving in different road conditions. More specifically, they performed simulated driving in urban and highway environments. Furthermore, along with the two main types of driving environments, the participants were asked to perform different secondary tasks. Such secondary tasks were designed with the objective of eliciting and combining cognitive, visual, and manual distractions.

### 2.2. Experimental Design and Protocol

**Figure 1:**
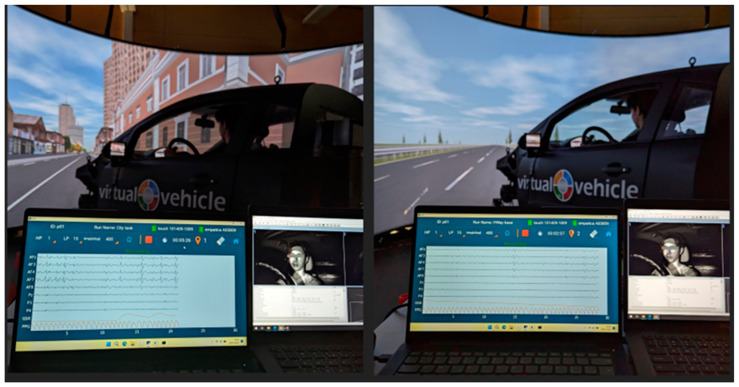
Experimental setting overview. On the left side, it can be observed the experimental settings related to the urban driving scenario (i.e., City), while on the right it is represented the non-urban driving scenario (i.e., Highway).

The experimental protocol was performed by employing a car simulator made of a real car structure, including its cockpit and seat, and a 140° high-definition screen (Virtual Vehicle, Graz, Austria). The experimental protocol consisted of two main environments, i.e., the “City” and the “Highway” environments, and different tasks. In Figure 1, an overview of the experimental settings is provided.

The driving simulator consisted of a cave system from VI grade with a full vehicle body and a 140° 3 times 4 k projector setup (Virtual Vehicle, Graz, Austria). The experimenter was located behind the participant outside of their view, and he was in constant communication with the participant via a two-way radio system (Virtual Vehicle, Graz, Austria). In addition to the three projectors showing the outside view of the vehicle, two small screens located left and right acted as rear mirrors. Immersion with the system was created using 5.1 surround sound (Creative, Singapore) for motor sounds and outside sounds, as well as four shakers on the axes of the car to simulate motor vibrations and road unevenness. The City and Highway scenarios were created in SCANeR by AVSimulation (v1.0). The experimental distractions were provided via a separate stereo sound system and via an Android tablet (Samsung, Seoul, South Korea) mounted to the right of the driver (to act as an infotainment), which was remotely controlled by the experimenter.

**Figure 2:**
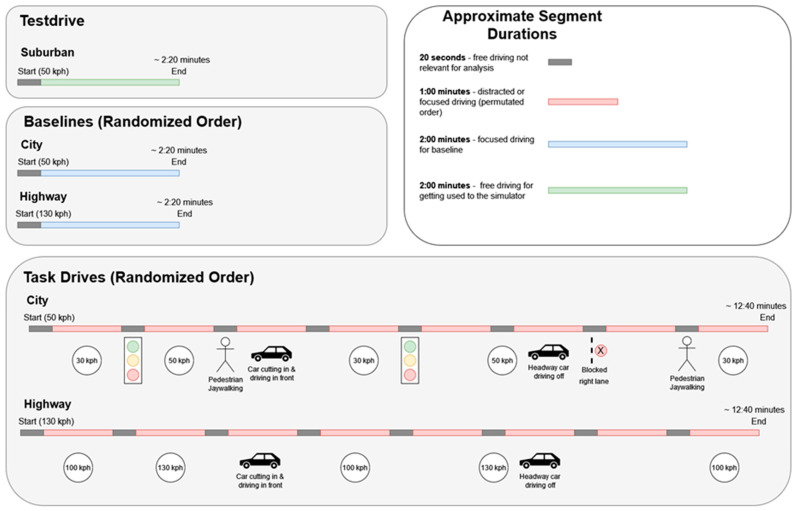
Experimental protocol overview.

The experimental protocol (Figure 2) included two baselines:

- City baseline: represented the focused driving task in an urban environment without any secondary tasks.
- Highway baseline: represented the focused driving task in a highway environment without any secondary tasks.

The two baselines were performed by the participants at the beginning of the experimental session. Subsequently, the participants were required to perform the two main sessions of the experimental protocol, i.e., the simulated driving in Highway and City environments. Each of them consisted of driving for almost 8 min in the given scenario (main task). At the same time, the main driving task was divided into segments (almost 60 s long) including specific requests and secondary tasks, alternated with segments of free driving without any additional request and/or task (named ***Other***) to reduce possible carry-over-effects between the task segments. The secondary tasks were selected in order to simulate the main distraction categories while driving, i.e., cognitive, cognitive, and visual, and visual and manual distractions. Such secondary tasks were identified as the following:

- ***Focused***: the participants were explicitly required to be focused while driving, without any secondary tasks.
- ***ACPT***: represented the Auditory Continuous Performance Task. This secondary task was specifically designed to elicit light cognitive distraction. Here participants were instructed to perform an auditory working memory task by listening to a series of auditory stimuli (i.e., randomized sequence of letters) and responding to a specific sequence of letters by answering orally while ignoring the other letters.
- ***Matrix***: represented the task in which the participants were asked to perform cognitively demanding pattern recognition and completion. This secondary task was designed specifically for eliciting cognitive and visual distraction. Participants were asked to identify the correct geometrical shape (i.e., circles) among a set of different shapes (i.e., circles, triangles, and squares) presented on the infotainment by providing oral feedback. Since participants did give their answers orally, the task did not induce manual distraction.
- ***SURT***: consisted of a visual search task in which participants must search for a slightly unique cue in a large set of similar cues. Participants were in fact asked to identify the slightly larger circle among a set of circles. Such a secondary task was designed for eliciting visual, and manual distraction because the participants had to indicate the unique cue by pressing their finger on the infotainment touch screen [33].

### 2.3. Subjective and Behavioral Data Collection and Analysis

To get an impression of the subjectively experienced distraction of the drivers, we provided a short questionnaire after their experimental session, where we asked for the perceived distraction in all four conditions, independently from the scenario (Highway and City). Participants were able to answer this on a seven-point Likert scale.

In addition to the subjective distraction data, we also assessed behavioral data associated with driver distraction with a SmartEye Pro eye tracker (Smart Eye AB, Gothenburg, Sweden) and by recording the driving behavior in the simulator. Analyzed eye tracking data consisting of the x and y position of the gaze vector. While driving data was primarily speed, steering wheel angle/torque, and acceleration. Because driving distraction is often the strongest represented in changes of behavioral regularity [34], we decided to analyze mostly the standard deviations of our dependent variables, and not the average values.

All subjective and behavioral data were analyzed by performing within subjects’ ANOVAs.

### 2.4. Neurophysiological Data Collection and Analysis

The drivers’ EEG signal was collected by the digital monitoring system Mindtooth Touch (BrainProducts GmbH, Gilching, Germany; https://mindtooth-eeg.com/, accessed on 14 February 2024) with a sampling frequency of 125 (Hz) [35,36]. The eight water-based recording electrodes were properly placed over the frontal and parietal brain areas commonly considered for mental state assessment [37,38]. In particular, the EEG channels were the following ones: AFz, AF3, AF4, AF7, AF8, Pz, P3, and P4, all referenced to the left mastoid and grounded to the right mastoid. All the electrodes’ impedances were kept below 50 (kΩ) and the quality of the EEG signals was checked before the experimental protocol started. The EEG headset was a wearable and comfortable device validated for applied research, with wireless connectivity in order to reduce as much as possible the interferences (the absence of wires means more naturalistic behavior for the user and more robust data) with the experimental setup.

Firstly, a band-pass filter with a 5th-order Butterworth within the interval of 2–30 (Hz) was applied to the EEG signal. Secondly, a notch filter centered on 50 (Hz) was applied for the AC noise-derived artifacts removal. The eye blink artifacts were detected and corrected online by a modified implementation of the Multi-Channel Wiener (MWF) [39] filtering through the Reblinca method [40]. More specifically, the ocular eye blink-based artifacts were detected through a statistical approach according to each participant’s eye blink pattern and, subsequently, corrected by applying a modified implementation of the MWF, which consisted of adaptive filtering. For further sources of artifacts, specific algorithms of the EEGLAB toolbox [41] were applied. Specifically, the pre-processed EEG signal has been divided into 1 s long epochs. Three criteria have been applied to recognize artifactual data automatically. Firstly, EEG epochs with a signal amplitude exceeding ±80 μV (Threshold criterion) were marked as “artifacts”. Then, each EEG epoch was interpolated to check the trend’s slope within the considered epoch (Trend estimation). If such a slope is higher than 20 μV/s, the considered epoch is marked as “artifact”. Finally, the signal sample-to-sample difference (Sample-to-sample criterion) was analyzed: if such a difference, in terms of absolute amplitude, was higher than 25 μV, i.e., an abrupt variation (no physiological) happened, the EEG epoch was marked as “artifact” [42,43]. In the end, the EEG epochs marked as “artifacts” were removed from the EEG dataset with the aim of having a clean EEG dataset to perform the analyses.

From the artifact-free EEG dataset, the Global Field Power (GFP) was calculated for the EEG frequency bands, namely Theta, Alpha, and Beta, needed for the mental states’ evaluation. The GFP was chosen as the parameter of interest describing brain EEG activity since it has the advantage of representing in the time domain the degree of synchronization of a specific cortical region of interest in a specific frequency band [44,45]. The EEG frequency bands were defined as a function of the Individual Alpha Frequency (IAF) value [46], i.e., the highest peak of the EEG spectrum within the traditional range of alpha rhythms (8–13 Hz). Since the Alpha peak is mainly prominent during rest conditions, the subjects were asked to keep their eyes open for a minute before starting the experiment. Such a condition was then used to estimate the IAF value specifically for each participant. In particular, the following frequency ranges have been adopted:

- Theta = [IAF − 8 ÷ IAF − 4] Hz;
- Alpha = [IAF − 2 ÷ IAF + 2] Hz;
- Beta = [IAF + 2 ÷ IAF + 20] Hz;

where the IAF individually computed for each participant was equal to 9.3 ± 1.8 Hz. It has to be noted that with IAF = 10 Hz, the bands range is equal to the standard definition.

The GFP was calculated over all the EEG channels for each epoch using a Hanning window of the same length of the considered epoch (1 s length, which means 1 Hz of frequency resolution), as the following:

$${GFP}_{band,region}=\frac{1}{N}\;\sum_{i=1}^{N}x_{i,band}^{2}(t),$$

where *N* is the number of the considered EEG channels and $x_{i,band}^{2}$ is the *i*-th EEG channel filtered within the selected EEG frequency band. After the EEG data preprocessing, the EEG GFP-derived features were computed to objectively characterize the relevant mental states within the above-described experimental protocol design. In particular, the mental workload, and the attention indexes were computed as follows:

$$Mental\;workload=\frac{Frontal\;Theta_{GFP}}{Parietal\;Alpha_{GFP}}=\frac{\frac{1}{5}\;\sum_{i=1}^{5}x_{i,theta}^{2}(t)}{\frac{1}{3}\;\sum_{i=1}^{3}x_{i,alpha}^{2}(t)},\;Attention=\frac{Frontal\;Beta_{GFP}}{Frontal\;Theta_{GFP}}=\frac{\frac{1}{5}\;\sum_{i=1}^{5}x_{i,beta}^{2}(t)}{\frac{1}{5}\;\sum_{i=1}^{5}x_{i,theta}^{2}(t)},$$

where $Frontal\;Theta_{GFP}$ and $Frontal\;Beta_{GFP}$ were computed by considering the AFz, AF3, AF4, AF7, and AF8 EEG channels, while the $Parietal\;Alpha_{GFP}$ was computed by considering the Pz, P3, and P4 EEG channels. In this regard, it has to be noted that computation of the mental workload index was defined according to the different previous work in which such mental state was deeply investigated as EEG-derived feature [47,48,49,50,51,52]. Similarly, the attention index definition was selected according to the inverse of the so-called Theta–Beta Ratio, an EEG-derived feature broadly validated as an ADHD indicator [53,54]. In this regard, it has to be highlighted that such a derivation of the index was previously validated in scientific literature as a neurophysiological representation of the distributed attention allocated to perform different simultaneous activities [55,56,57].

The computation of these two specific indicators for the definition of the proposed EEG-based distraction index derives from the psychological definition of distraction components described in the Introduction. In particular, our assumption is that, if the user is fully engaged in the main task, it should experience a certain level of mental workload jointly with a high level of sustained attention. On the other side, in a “Distracted” condition, the user would experience an even higher level of workload (because of concurrent secondary tasks or mind wondering) but with lower levels of sustained attention.

Therefore, the Human Distraction Index (*HDI*) was defined by combining the two above defined mental states, i.e., the mental workload and the attention, as follows:

$$HDI=Mental\;workload-Attention=\frac{Frontal\;Theta_{GFP}}{Parietal\;Alpha_{GFP}}-\frac{Frontal\;Beta_{GFP}}{Frontal\;Theta_{GFP}}$$

### 2.5. Statistical Analyses

The statistical analyses were conducted following the data normalization. More specifically, the subjective and behavioral data were not normalized since the self-reported distraction scores were already provided through a standard seven-point Likert scale, while the standard deviation of the driving parameters distributions were considered within the presented analyses. Meanwhile, the EEG features, i.e., the HDI, was subjected to normalization using the z-score technique, referencing the respective baseline collected within the City and Highway driving scenario. Subsequently, the Shapiro–Wilk test was used to assess the normality of the distribution related to each of the considered parameters [58]. If normality was confirmed, the Student’s *t*-test was performed for the group comparison (i.e., City vs. Highway). In the case of non-normal distribution, the Mann–Whitney test was performed. In case of comparisons between three or more distributions, the analysis of variance (ANOVA) or its non-parametric equivalent (Friedman ANOVA) was performed. For all tests, statistical significance was set at α = 0.05.

## 3. Results

This section was divided into different subparagraphs in order to organize all the results on the basis of the related data source, i.e., questionnaires (subjective results), eye tracking and driving parameters (behavioral results), and neurophysiological ones.

Among all the comparisons, only the analysis providing statistically significant results are here reported.

### 3.1. Subjective Results

**Figure 3:**
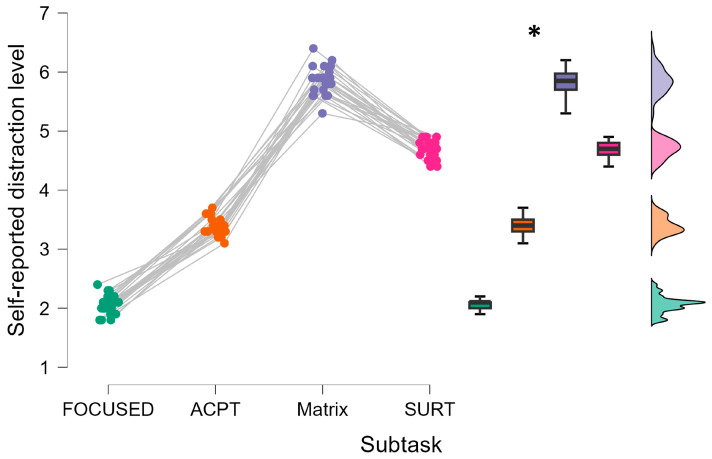
The participants perceived the Matrix and SURT subtasks as the more distractive while driving. Focused driving was perceived as less distracting. * represents the statistical differences observed within the tested distributions. Each color refers to the data distribution collected in each specific driving subtask.

The statistical analysis revealed that the subjective distraction level differed significantly between all types of subtasks (F = 79.4, *p* < 0.001, η^2^ = 0.672). As expected, the distraction level for the focused group was lower than for all other groups (Figure 3). In particular, the post-hoc statistical tests showed that Matrix and SURT subtasks were perceived as the more distractive while driving (all *p* < 0.001).

### 3.2. Behavioral Results

**Figure 4:**
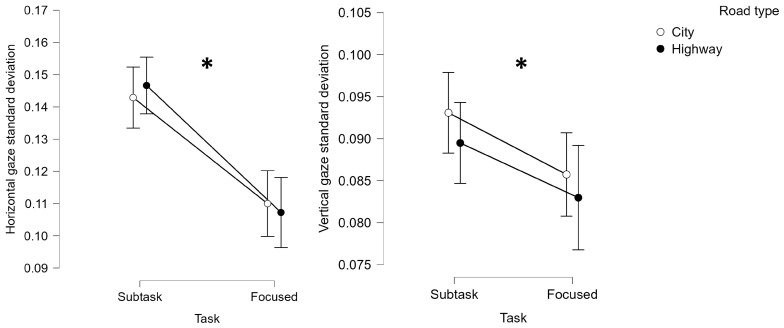
The eye-tracking derived parameters, i.e., the horizontal and vertical eye gaze, were characterized by a significantly higher standard deviations when the participants drove while performing a secondary task (i.e., Subtask) compared to their focused driving (i.e., Focused). * represents the statistical differences observed within the tested distributions.

Regarding the eye-tracking derived parameters, i.e., the horizontal and vertical eye gazes’ standard deviations, the statistical analyses demonstrated a significant difference between the focused and the non-focused, i.e., the overall ACPT, Matrix, and SURT driving segments (Subtask), experimental phases (Horizontal gaze: Friedman chi-squared = 63.176, *p* < 0.001, η^2^ = 0.588); Vertical gaze: Friedman chi-squared = 4.485, *p* = 0.04, η^2^ = 0.449). More specifically, the horizontal and vertical eye gazes resulted to be significantly unregular when performing secondary tasks, i.e., Subtask, while driving compared to the focused driving (Figure 4).

**Figure 5:**
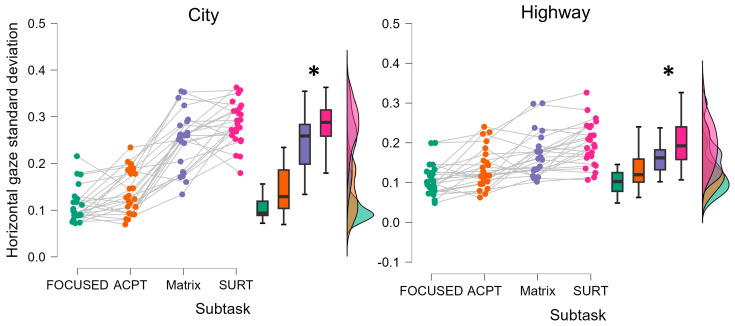
The horizontal gaze standard deviation resulted to be significantly higher during the Matrix and SURT subtasks compared to the others, both in City and Highway driving scenarios. * represents the statistical differences observed within the tested distributions.

Moreover, the Friedman ANOVA revealed that both in City and Highway scenario the horizontal gaze standard deviation was significantly higher during the Matrix and SURT subtasks compared to the others (City: Friedman chi-squared = 117.558, *p* < 0.001, η^2^ = 0.512; Highway: Friedman chi-squared = 37.858, *p* < 0.001, η^2^ = 0.509), representing a significant higher distraction during such two driving subtasks (Figure 5).

**Figure 6:**
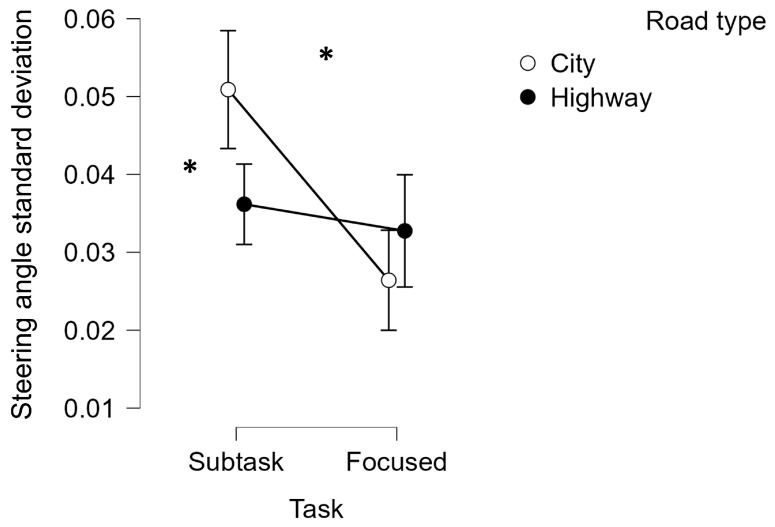
Steering angle standard deviation resulted in statistically higher (*p* < 0.001) while driving and performing secondary tasks, especially within the City scenario compared to the Highway one. * represents the statistical differences observed within the tested distributions.

Regarding the statistical analysis performed on the behavioral parameters related to driving, the standard deviations of the steering angle, speed, and acceleration distributions were considered. The Friedman ANOVA performed on the steering angle standard deviation revealed a significant statistical effect of the subtask request (i.e., Subtask) (Friedman chi-squared = 26.472, *p* < 0.001, η^2^ = 0.651) and a combined statistical effect of the subtask request and the road type (Friedman chi-squared = 16.268, *p* < 0.001, η^2^ = 0.691). This result represented how the participants drove with a lower grade of stability within the City scenario while performing secondary driving tasks. Any statistical differences were observed among the different subtasks within the two driving scenarios in terms of steering angle standard deviation (Figure 6).

**Figure 7:**
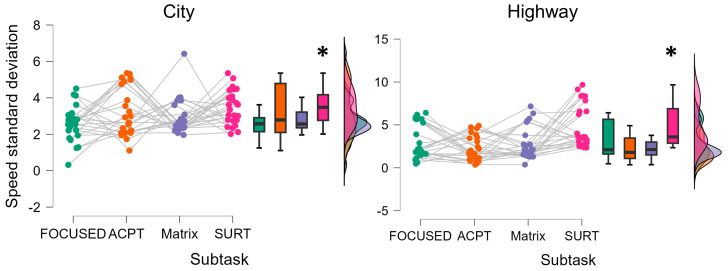
The Friedman ANOVA showed how the speed standard deviation statistically increased during the SURT subtask compared to the others, both in City (*p* = 0.04) and Highway scenarios (*p* < 0.001). * represents the statistical differences observed within the tested distributions.

The Friedman ANOVA performed on the speed standard deviation revealed that this driving parameter statistically increased when performing the SURT subtask compared to the others both within the City and Highway scenarios (City: Friedman chi-squared = 2.807, *p* = 0.04, η^2^ = 0.487; Highway: Friedman chi-squared = 6.280, *p* < 0.001, η^2^ = 0.527) (Figure 7). In this regard, no statistical differences were observed in terms of speed standard deviation between City and Highway scenarios.

**Figure 8:**
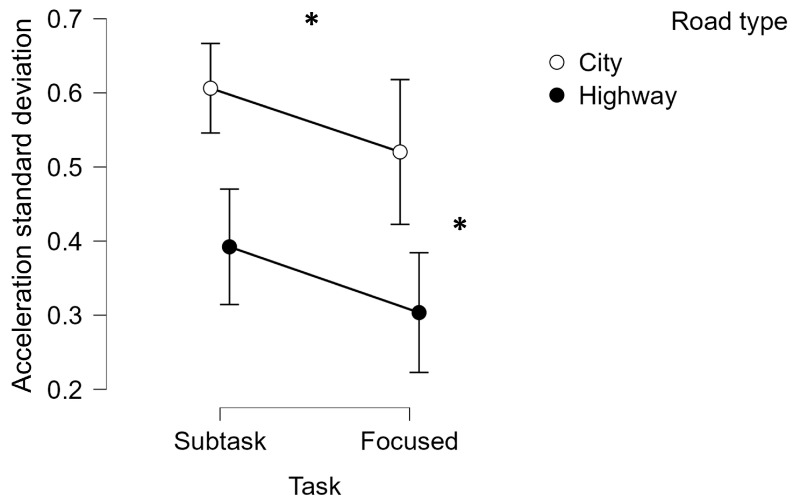
The acceleration standard deviation resulted in being significantly higher when performing a secondary task while driving (i.e., the Subtask condition) compared to the focused driving (*p* < 0.001). Furthermore, the acceleration standard deviation statistically increased while driving in City scenarios compared to the Highway ones (*p* < 0.001). * represents the statistical differences observed within the tested distributions.

Finally, the statistical analysis performed on the acceleration standard deviation highlighted how such a parameter statistically increased when driving by performing a secondary task and driving within the City scenario compared to the Highway one (Subtask: Friedman chi-squared = 6.190, *p* = 0.02, η^2^ = 0.408; road type: Friedman chi-squared = 20.630, *p* < 0.001, η^2^ = 0.639) (Figure 8). No statistical differences were highlighted when comparing the different subtasks within the two driving scenarios (i.e., City and Highway).

### 3.3. Neurophysiological Results: EEG-Based Distraction Index

**Figure 9:**
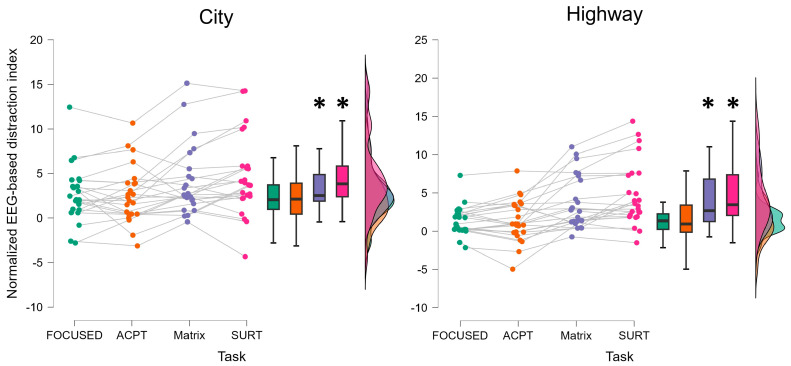
Normalized EEG-based distraction index evaluated along the different subtasks within the City driving environment. The statistical analyses revealed that the Matrix and SURT subtasks resulted to be significantly more distractive within both the two driving scenarios. * represents the statistical differences observed within the tested distributions.

The Friedman statistical test performed on EEG-based distraction index distribution evaluated along the different subtasks completed within the City simulated driving scenario showed a main statistical effect related to the secondary driving tasks (Friedman chi-squared = 6.487, *p* < 0.001, η^2^ = 0.708). The post-hoc analysis revealed that during the SURT subtask the measured distraction was statistically higher compared to the condition in which the participants did not perform any secondary task (i.e., FOCUSED) and during the one in which only auditory stimuli was provided (i.e., ACPT) (*p* < 0.02) (Figure 9).

Similarly, the Friedman test performed on the EEG-based distraction evaluations within the Highway simulated driving environment revealed a statistically significant main effect of the secondary tasks (Friedman chi-squared = 18.250; *p* < 0.001, η^2^ = 0.772). More specifically, the post-hoc analysis showed that the drivers’ distraction resulted to be significantly higher during the MATRIX and SURT subtasks compared to the FOCUSED and ACPT ones (all *p* < 0.001) (Figure 9).

Moreover, a second type of statistical analysis was performed in order to compare the EEG-based distraction index overall evaluated within the City and Highway simulated driving environments. More specifically, the distraction EEG-derived index was compared between the City and Highway driving conditions by averaging such index along all the subtasks in which a secondary activity was required, i.e., ACPT, Matrix, and SURT, defined as Subtask. Similarly, the subtask in which the participants were required to perform just a focused driving within the two simulated driving environments, i.e., Focused, were compared in terms of drivers’ distraction variations.

**Figure 10:**
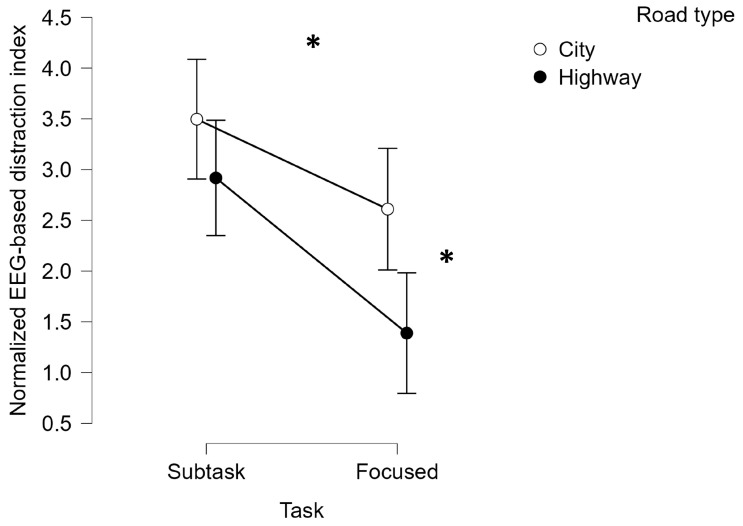
Normalized EEG-based distraction index averaged along all the subtasks and within the focused driving condition. The statistical analysis revealed that the drivers were more distracted during the secondary task (i.e., Subtask) execution and within the simulated driving in urban environments (i.e., City). * represents the statistical differences observed within the tested distributions.

In particular, Figure 10 shows that the statistical analysis performed on the EEG-based distraction index revealed a statistical effect of the secondary execution compared to the focused driving, and a statistical effect of the road type (Subtask: Friedman chi-squared = 12.849, *p* = 0.002, η^2^ = 0.618; Road type: Friedman chi-squared = 8.910, *p* = 0.01, η^2^ = 0.491). More interestingly, the statistical analysis showed a statistically significant interaction between the subtask request and the road type (Friedman chi-squared = 5.942, *p* = 0.03, η^2^ = 0.446).

**Figure 11:**
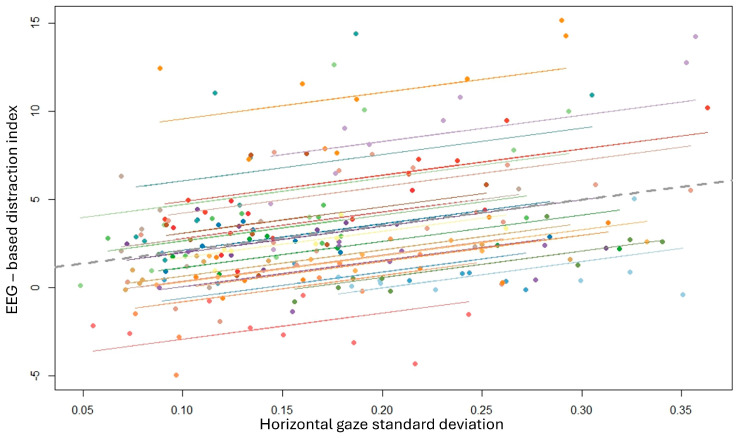
The repeated measure correlation analysis demonstrated a positive and significant correlation between the EEG-based distraction index and the horizontal gaze standard deviation among all the participants. Each line represents the correlation estimated for each single participant, as well as the same color dots are referred to the data distribution for each specific participant.

Finally, a final statistical analysis was performed to assess the correlation between the proposed EEG-based distraction index and the behavioral measurements collected for the distraction assessment. In particular, the repeated measure correlation analysis [59] was conducted between the EEG-based distraction index and ocular gaze (i.e., horizontal and vertical), the speed, and steering wheel standard deviations. The statistical results demonstrated that the EEG-based distraction index was positively and significantly correlated only with the horizontal gaze standard deviation (R = 0.58, *p* < 10^−12^) (Figure 11).

## 4. Discussion

The objective of the presented study was focused on the development of an objective indicator for evaluating the drivers’ distraction, relying on the EEG signal collected while driving in a simulated environment. The reliability of this proposed EEG-based distraction index was investigated through an experimental protocol designed for modulating the drivers’ attentional demand by requesting secondary tasks while driving. Such secondary tasks were compared with the condition with only the main task, i.e., the focused driving. Also, the drivers were performing the same simulated driving within both an urban (i.e., City) and a non-urban (i.e., Highway) scenario. Therefore, the drivers’ distraction modulations were assessed according to two dimensions: (i) the secondary tasks’ impact and (ii) the road type’s impact.

The drivers’ EEG signal was collected along the entire experimental protocol coupled with subjective and behavioral data. In particular, the distraction self-evaluations provided by the drivers at the end of each subtask were considered as subjective measurements, while vehicular data and eye tracking data were considered along the analysis as behavioral data. Concerning the secondary task impact, it was hypothesized that the ACPT, i.e., the subtask in which the drivers were asked to answer only to a specific audio stimulus, and the FOCUSED, i.e., the condition in which the drivers were required to perform a focused driving without any additional secondary activities were the less distractive subtasks. While the MATRIX, i.e., the subtask designed for eliciting cognitive and visual distraction, and the SURT, i.e., the subtask inducing visual and manual distraction, were hypothesized to be more distractive. Such experimental hypotheses were significantly validated through the analysis of the subjective and behavioral measurements. The distraction self-evaluations demonstrated that the drivers perceived the Matrix and SURT as the more distractive subtasks while driving. Regarding the behavioral measurements associated with the drivers’ ocular patterns, the statistical analysis revealed that during the Matrix and SURT, the horizontal gaze variance was significantly higher compared to the others. In this regard, previous scientific works largely demonstrated that such a parameter (i.e., ocular gaze variance) is associated with the tunnel view effect due to cognitive and visual distraction [60,61,62] (REF). Therefore, the abovementioned results can be related to a significant distraction increase while performing the Matrix and SURT subtasks while driving. This was confirmed also by the vehicular data analysis. According to the related scientific literature, the statistical analysis revealed that distracted drivers generally tend to vary their speed and acceleration more consistently. Similarly, the analysis demonstrated that during the more distractive secondary driving activities (i.e., ACPT, Matrix, and SURT), the drivers showed less regularity in their steering behavior.

Regarding the second dimension assessed along the presented analysis, i.e., the road type impact on the drivers’ distraction, the drivers’ ocular patterns and the vehicular data (i.e., speed and acceleration standard deviation) revealed that during the driving activities performed within an urban simulated scenario (i.e., City scenario), the drivers appeared to be more distracted.

Taken together, the statistical results associated with the subjective and behavioral data validated the experimental hypothesis, for which the drivers’ distraction increases while performing secondary tasks within the simulated driving and, moreover, the drivers’ distraction increases when performing the simulated driving within an urban scenario.

Concerning the reliability assessment of the proposed EEG-based distraction index, the presented results revealed that such a synthetic index allowed to capture of the drivers’ distraction variations along the two aforementioned dimensions, i.e., the secondary tasks execution while driving, and the road type’s impact. In fact, the EEG-based distraction index resulted significantly higher when evaluated along the MATRIX and SURT experimental conditions, in which the drivers experienced cognitive, visual, and manual distractions while driving. This was observed within both the urban and non-urban scenarios. More importantly, the EEG-based distraction index exhibited significant reliability in detecting the drivers’ distraction variations between the two proposed urban and non-urban scenarios, resulting in significantly higher within the City scenario. Such a result can be explained by observing that the drivers were required to handle several distracting elements within the urban scenario with respect to the Highway one. In particular, such distracting elements, such as pedestrians, other cars’ crossings, streetlights, etc., are “naturally” integrated within the driving scenario. In other words, the proposed EEG-based distraction index demonstrated to be reliable in capturing different categories of distracting elements: (i) the cognitive, visual, and manual distraction induced by the execution of secondary activities while driving, and (ii) the cognitive and visual distraction induced by the driving scenario itself. Such an aspect is particularly promising with respect to future applications and developments of the proposed index. In this regard, it has to be noted that the proposed EEG-based distraction index corresponds to a unique indicator which was demonstrated to be sensible to different declinations of the drivers’ distraction as much as the combination of the several subjective and behavioral parameters presented within this study. The proposed index does not require the installation of ocular pattern detectors, as well as it does not require the collection and processing of several vehicular data for computing an objective drivers’ distraction evaluation. More importantly, the proposed EEG-based distraction index is fully compatible with an online implementation, which could allow to timely detect or even prevent the drivers’ distraction while driving.

Indeed, there are limitations to be discussed. First of all, even trying to have a sample as much homogeneous as possible, it resulted difficult to achieve this objective in terms of age. It is demonstrated that age impacts brain activity patterns. At the same time, the study does not have a sufficient sample size to perform a subgroup analysis (by using age as a grouping variable). In terms of gender, no statistical differences were found among participants. Additionally, confounding variables were implicitly included in the presented protocol, such as the motion sickness deriving from the driving simulator usage, and the different confidence levels with the simulator dashboard for the secondary tasks’ execution. In any case, because of the novelty of the outcomes and the exploratory essence of the study, we did not consider it as a critical concern. However, in future steps, we will perform and suggest follow-up studies aimed at validating these preliminary results on a more homogeneous sample. The presented experimental protocol was designed to induce different distraction grades and it can be argued that the proposed index would solely be sensible to specific and emphasized driving distraction conditions. However, the present study aimed to validate the reliability of a synthetic EEG-based index in detecting driving distraction. A future step will consist of the application of such an approach in a more realistic context. In this regard, it has to be observed that the presented EEG-based distraction index could be further tested on reduced EEG channel configurations, in order to assess its reliability on wearable EEG system even lighter than the one used for the presented research. In the same context, the proposed EEG-based index could be selected as a benchmark, given its high time resolution, for assessing its correlation with other neurophysiological and autonomic-derived features, such as the ones driving the cardiac activity and the electrodermal activity.

## 5. Conclusions

The current investigation, employing a simulated driving protocol, aimed at developing and validating an innovative EEG-based index for detecting driving distraction. Results demonstrated the reliability of the proposed EEG-based distraction index in identifying the drivers’ distraction while driving along different conditions, representing a synthetic, objective, and more ecological solution with respect to the largely validated subjective and behavioral measurements. More importantly, the technical implementation of the proposed index allows for the driving distraction online evaluation while driving, even in real context since the proposed index was derived from EEG signal collected through a wearable EEG system, which was demonstrated to be reliable and compatible with out-of-the-lab applications [35]. In summary, this study, alongside its findings, lays the groundwork for integrating neurophysiological evaluation of driving distraction into real-world settings to enhance road traffic safety, as well as a powerful tool for further investigation of the driver’s psychology and behavior.

## Data Availability

The data that support the findings of this study are available from the corresponding authors upon reasonable request. The data are not publicly available since they are biometric data and they are considered as sensitive data as of EU GDPR n. 2016/679.
